# Peptide-Based Biomaterials for Combatting Infections and Improving Drug Delivery

**DOI:** 10.3390/pharmaceutics16111468

**Published:** 2024-11-18

**Authors:** Lucia Lombardi, Jiaxu Li, Daryl R. Williams

**Affiliations:** Department of Chemical Engineering, South Kensington Campus, Imperial College London, London SW7 2AZ, UK

**Keywords:** self-assembling peptides, implant coating, antimicrobial gels, drug delivery, peptoid sheet

## Abstract

This review explores the potential of peptide-based biomaterials to enhance biomedical applications through self-assembly, biological responsiveness, and selective targeting. Peptides are presented as versatile agents for antimicrobial activity and drug delivery, with recent approaches incorporating antimicrobial peptides into self-assembling systems to improve effectiveness and reduce resistance. The review also covers peptide-based nanocarriers for cancer drug delivery, highlighting their improved stability, targeted delivery, and reduced side effects. The focus of this work is on the bioactive properties of peptides, particularly in infection control and drug delivery, rather than on their structural design or material characteristics. Additionally, it examines the role of peptidomimetics in broadening biomaterial applications and enhancing resistance to enzymatic degradation. Finally, the review discusses the commercial prospects and challenges of translating peptide biomaterials into clinical applications.

## 1. Introduction

Peptides, derived from natural or synthetic amino acids, represent fundamental building blocks for constructing complex molecular assemblies [1]. Their inherent capacity for self-assembly makes them excellent candidates for the development of smart biomaterials with well-defined architectures and diverse functionalities responsive to various environmental stimuli. Peptide self-assembly is predominantly driven by non-covalent interactions, which can be finely tuned by altering amino acid sequences and modulating environmental conditions [2]. These interactions enable precise control over the formation of a wide array of supramolecular nanostructures, including nanotubes, nanobelts, fibrils, nanovesicles, gels, and nanocages [3,4]. The transition from peptide-based nanostructures, such as nanotubes, fibrils, and vesicles, to hydrogels involves their self-assembly into three-dimensional networks capable of encapsulating water molecules. This transformation relies on the inherent physical properties and intermolecular interactions of the peptides, which can be tailored by adjusting chemical and environmental parameters. These hydrogels have demonstrated significant potential in various biomedical applications due to their unique physicochemical properties and biocompatibility.

A key advantage of peptide biomaterials lies in their ability to emulate the extracellular matrix (ECM), offering structural support and bioactive signals that promote cell adhesion, proliferation, and differentiation [5]. By designing specific peptide sequences, bioactive surfaces can be engineered to interact with integrin receptors, facilitating favourable cellular responses. Additionally, peptides can be programmed to assemble into diverse supramolecular structures capable of dynamic disassembly in response to specific biological triggers [6]. This feature allows for the controlled release of encapsulated therapeutics or targeted disruption of biological processes, such as biofilm formation. There are two primary strategies for synthesizing self-assembling peptides: solid-phase peptide synthesis (SPPS) and protein engineering [7,8]. SPPS is widely used to produce short to medium-length peptide sequences (up to 70 amino acids), while protein engineering enables the generation of longer well-defined peptides, such as collagen- and silk-inspired sequences, using bacterial expression systems. Recent advancements in peptide synthesis, computational modeling, and high-throughput screening have catalysed the development of innovative peptide biomaterials with enhanced properties [9,10]. These advancements have facilitated the design of materials that respond to specific physiological conditions, such as pH, temperature, or enzymatic activity, enabling targeted therapeutic delivery and dynamic interactions with the biological environment. Despite these promising developments, challenges remain, including enhancing stability, scalability, and the precise tuning of bioactivity for specific clinical applications.

While numerous reviews on peptide-based biomaterials have been published, they often adopt a broad structural perspective, focusing primarily on mechanical properties, design innovations, and [3,11] material characteristics rather than on the bioactive peptide-based strategies [12] and peptide applications in wound healing and regenerative medicine, prioritising tissue repair over infection prevention or drug delivery [13].

This review aims to address these gaps by highlighting the dual functionality of peptide-based biomaterials as both antimicrobial agents and drug delivery systems, showcasing the versatility of peptides in addressing complex healthcare challenges. It also explores the potential of peptidomimetics to expand the range of biomaterials and enhance resistance to enzymatic degradation, which is crucial for drug delivery applications and peptide-based therapeutics. Furthermore, this review examines the translational potential of peptide biomaterials, offering insights into their pathways toward commercialisation.

## 2. Classification of Self-Assembled Peptides

**Figure 1:**
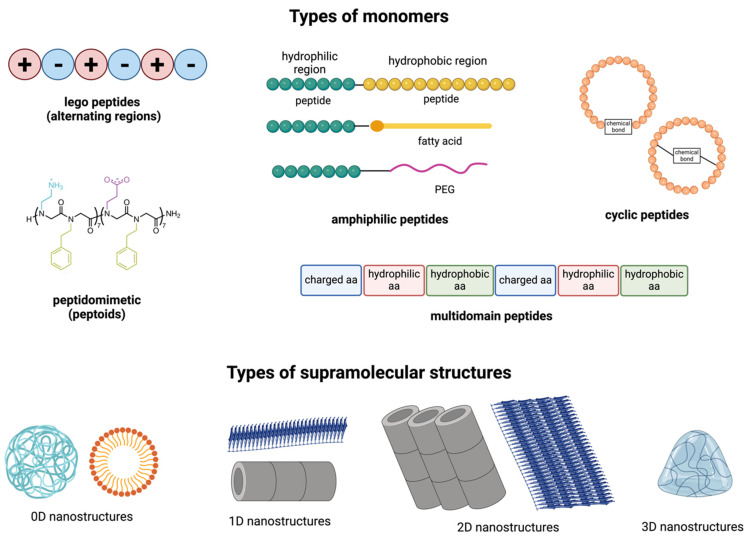
Main categories of peptide monomers that can form biomaterials by self-aggregation. According to the monomers and the interactions between monomers, the self-assembled structure can be a sphere (0D nanostructure), a tube (1D), a fibre (1D), a sheet (2D), or a hydrogel (3D). Created with Biorender.com.

Peptide self-assembly can occur spontaneously or be induced under specific solution conditions. Like other macromolecules, peptide self-assembly predominantly relies on non-covalent intermolecular interactions. The driving forces behind peptide self-assembly are primarily determined by the physico-chemical characteristics of the amino acids within the peptide and its sequence. These forces include hydrogen bonding, π–π stacking, hydrophobic interactions, electrostatic interactions, and van der Waals forces and often coexist within aggregates, with certain forces dominating [14]. Broadly, peptides can be classified into different categories as outlined below (Figure 1 and Table 1).

### 2.1. Ionic Peptides

Ionic peptides are a class of peptides with an alternating arrangement of negatively and positively charged residues, leading to electrostatic interactions that drive self-assembly, along with hydrogen bonding and van der Waals forces. These peptides can form stable β-sheet structures in aqueous solutions, making them valuable in designing biomaterials that mimic the natural extracellular matrix (ECM). Due to their pure amino acid composition and sol-gel transition properties, they have gained significant attention in biomaterials science and regenerative medicine [30]. For instance, Yang et al. used the ionic complementary peptide EFK16-II to modify electrodes for glucose biosensing, demonstrating its potential in biocompatible interfaces for molecular sensing [31]. Wan et al. studied EAK16-II-derived peptides, revealing that peptide length and the amino acid arrangements influence the morphology and anticancer activity of peptide–drug complexes [32]. 

### 2.2. Amphiphilic Peptides

Peptide amphiphiles (PAs) consist of hydrophilic bioactive sequences linked to hydrophobic regions, such as fatty acid lipids, hydrophobic amino acid chains, or synthetic polymers. These peptides self-assemble into structures with specific morphologies and sizes driven by intermolecular hydrophobic interactions [17,33]. Diverse structures of proteins across different length scales, from intrachain folding to the complex assembly of multiple protein units, are driven by the precise sequences of amino acids. Similarly, PAs, which consist of peptides coupled to synthetic hydrophobic tails, mimic this versatility by offering tuneable structures and multichain assembly behaviour. Synthetic methods, including solid-phase peptide synthesis, enable precise control over amino acid sequences, allowing for the strategic placement of functional groups. Typically, PAs feature diblock or triblock structures with both hydrophobic and hydrophilic segments.

Amphiphilic peptides can be further divided into some subcategories, including surfactant-like peptides, bolaamphiphilic peptides, and lipidated peptides, depending on the number of hydrophilic groups, and the composition of the hydrophobic region. Surfactant-like peptides contain either positively charged or negatively charged amino acids in the hydrophilic head linked to the hydrophobic tail, such as Ac-A_6_K_2_, Ac-V_6_K, and Ac-A_6_D [34,35]. This structure enables the peptides to self-assemble in aqueous solution with hydrophobic groups orientating away from the water molecules in the formation of different nanostructures. In contrast to surfactant-like peptides, which typically feature one single polar head group in the structure, bolaamphiphilic peptides, or bolaforms, are characterised by the presence of two polar head groups connected by a hydrophobic spacer. Lipidated self-assembling peptides contain peptide sequences modified with hydrophobic lipid chains, which facilitate the formation of secondary structures and enable the self-assembly into a range of well-defined morphologies. The most commonly investigated lipidated peptides feature a single alkyl chain attached to the N-terminus, which allows for the attachment of various bioactive ligands at the C-terminus, expanding their functional applications [29,36]. For instance, lipopeptides C_16_IKPEAP and C_16_IKPEAPG, which are derived from the gastrointestinal peptide hormone PYY3−36, can self-assemble into micelles and further develop into β-sheet amyloid fibrils when deprived of water [37]. 

Similar to traditional surfactants and lipids, many of these amphiphilic peptides also have a defined critical aggregation concentration (CAC), above which the peptides will self-assemble into various nanostructures depending on solution pH, temperature, and ionic strength [38,39]. For example, Mondal et al. designed a cationic bolaform, which formed elongated micelles with a relatively low CAC due to its hydroxyl functionality and demonstrated good gene transfection efficiency despite their strong DNA-binding affinity [18].

### 2.3. Peptidomimetics

Synthetic peptides or peptidomimetics, including peptoids, are gaining significant attention in materials science, nanotechnology, medicine, and biomedical engineering for their stable and intriguing self-assembled structures.

The synthesis of functional two-dimensional (2D) nanomaterials, especially polymer- and protein-based varieties, is gaining prominence across diverse applications. Peptoids, which are protein-mimetic polymers, exhibit potential for constructing 2D nanostructures applicable in molecular recognition, catalysis, membranes, and nanoparticle assembly. Recently discovered peptoid nanosheets form through distinctive monolayer collapse mechanisms at either the air–water or the oil–water interface. Chemical modifications to the peptoid sequence affect nanosheet formation, presenting challenges in maintaining the necessary hydrophobicity for interfacial adsorption. Peptoids featuring alternative sequences of ionic and hydrophobic monomers that spontaneously adsorb at the liquid–air interface create a highly ordered monolayer intermediate in dilute aqueous solution. The compression of these surface layers induces monolayer buckling and collapse into a bilayer in the aqueous phase, with the hydrophobic groups forming an interior core and ionic groups exposed on the surface. To assess the chemical modification tolerance for nanosheet formation, the Zuckermann group systematically examined chemical analogues of prototype sheet-forming peptoids. Decreasing the peptoid hydrophobicity may disrupt nanosheet formation, affecting interfacial adsorption, while an increase in peptoid hydrophobicity may result in unwanted aggregation. Recent studies, however, reveal that the ability to form a monolayer is not the sole requirement for nanosheet formation [40].

### 2.4. Cyclic Peptides

Cyclic peptides are commonly found in natural products, sometimes offering a range of pharmacological activities. Compared to linear peptides, cyclic peptides often show higher binding affinity, improved protease stability, and enhanced cellular uptake. They can be classified based on their origin, physicochemical properties, and conformation. Zhang et al. demonstrated the efficient synthesis of cyclic peptides using ortho-phthalaldehyde (OPA)-amine-thiol reactions, offering a new tool for constructing DNA-encoded peptide libraries [41]. Shirazi et al. synthesised a peptide-based cyclic [WH]5 drug delivery system that improved the cellular uptake of cell-impermeable cargo [42]. 

### 2.5. Multidomain Peptides

Multidomain peptides (MDPs) consist of hydrophilic, hydrophobic, and charged regions that can drive their self-assembly into nanofibers by eliminating water from the hydrophobic core and forming hydrogen-bonding networks [43]. Their structural properties can be tuned by manipulating the electrostatic interactions between the peptides, including changes in the solution ionic strength, pH, and salt concentration of the solvent, allowing the formation of hydrogels that are shear recoverable [6,44]. MDPs have demonstrated antimicrobial properties and can be tailored to exhibit a range of biological functions through structural modifications. For instance, Wickremasinghe et al. demonstrated that MDP nanofibers, when combined with growth-factor-loaded liposomes, can create composite hydrogels that enable the time-controlled release of placental growth factor-1 (PlGF-1) [45]. It was demonstrated that these MDP-liposome hydrogels offered enhanced angiogenic responses and promoted vessel regeneration, offering a biocompatible platform for drug delivery and potential treatments for ischemic tissue diseases.

The classification provided above is a broad approach to categorising self-assembled peptides, primarily based on structural attributes such as hydrophilic, hydrophobic, and charged domains. However, numerous other classification systems exist due to the overlapping and complex characteristics and multifunctional behaviours of SAPs. For instance, SAPs can also be grouped according to their self-assembly mechanisms, peptide length, or biofunctional roles [46]

## 3. Different Types of Self-Assembled Nanostructures

Peptides can self-assemble into diverse hierarchical structures, including 0D nanoparticles and 1D, 2D, and 3D conformations (Figure 1) [1]. In contrast to bulk materials, which can exhibit three-dimensional characteristics such as height, length, and width, 0D nanostructures are individual particles or clusters without these three canonical dimensions. Due to their small size and high surface-to-volume ratio, they offer many reactive sites per unit mass, making them ideal for nanomaterial applications [47]. Such self-assembled peptide nanoparticles and nanospheres are common examples of 0D nanostructures, typically formed through noncovalent π–π interactions, van der Waals, and hydrophobic forces between different peptide sequences [48]. Hydrophobic interactions are crucial in the self-assembly of these spherical nanoparticles. Following the principles of entropy minimisation and stability, the hydrophobic regions of amphiphiles aggregate into a core. At the same time, hydrophilic segments arrange themselves on the outer surface to form a shell-like morphology, thereby enhancing their interactions with water [49].

Amyloid fibrils represent a well-characterized example of a 1D self-assembly structure, where peptides form nanofibrillar structures characterised by β-sheet-like secondary structures [50]. Amyloids, which are aggregates of peptides or proteins, result from the oligomerisation of amyloidogenic sequences and are often associated with degenerative and chronic diseases [51]. A minimalistic model developed by the Gazit group demonstrated the self-assembly of diphenylalanine (FF), the shortest functional unit, into nanofibrillar structures [52]. Furthermore, the non-destructive self-assembly of Y-rich peptide nanofibers was conjugated with silver nanoparticles and carbon nanotubes to create functional nanoelectrodes [53]. Similarly, Cormier et al. explored the self-healing properties of nanofibers formed from the peptide RADA16-1. In this case, alternating hydrophobic and charged subunits led to the formation of β-strands, creating a hydrophobic core and hydrophilic surface, with two β-strands stacking into the fundamental fibril unit [54].

Two-dimensional nanostructures have a planar lattice geometry extending in two dimensions. The inherent chirality of amino acids facilitates 1D elongation while limiting lateral growth, with peptide helices often employed to generate 2D morphologies [26]. The self-assembly of heptapeptide KLVFFAK resulted in amyloid 2D nanosheets, as distinct from traditional amyloid fibrils, and was investigated in aqueous solutions. The study revealed a two-dimensional expansion along the fibril axis and a perpendicular “zippering” axis. In classic amyloid fibril assembly, β-sheets extend through hydrogen bonding, while perpendicularly arranged β-sheets pair into “steric zippers” with hydrophobic interfaces formed by side chains [27]. Lee et al. identified a peptide sequence (YFCFY) that underwent a transition from monomer to dimer via disulfide bridge formation between cysteine subunits. This disulfide linkage initiated and stabilised helix formation, resulting in 2D macroscopic flat sheets [55].

Peptides can also form 3D structures like hydrogels, commonly used in drug delivery and wound healing. The formation of these hydrogels depends on a balance between hydrophobic and hydrophilic interactions and the presence of aromatic residues [56]. Various secondary structures contribute to this hierarchical assembly, including β-pleated sheets and α-helices. These hydrogels can offer stimuli-responsive properties (e.g., pH and enzyme responsiveness), enabling targeted drug delivery. For example, a pH-responsive octapeptide, FOE, can self-assemble into a hydrogel at physiological pH, displaying injectability and anticancer properties [57]. Peptide hydrogels, especially β-peptide hydrogels, have shown promise in several applications in tissue engineering due to their enhanced metabolic stability.

## 4. Hybrid Materials

Substantial progress has been achieved in conjugating peptides to various biomaterials, significantly enhancing their functional properties. Peptides offer several advantages, including high specificity, potency, cost-effectiveness, small size for improved tissue penetration and targeted delivery, biodegradability, and novel therapeutic potential. By incorporating these unique features into biomaterials, numerous advancements have been made. Among the various methods used for biomaterials modification, chemical techniques are the most well-established, providing an efficient means to immobilise target molecules onto specific surfaces.

Bioconjugation is a chemical process that links two substances through covalent or non-covalent bonds. Covalent bioconjugation is a preferred approach due to the stability of the bond and, generally, requires more stringent conditions than molecule organic synthesis. For instance, it is essential to maintain mild reaction conditions such as temperatures below 37 °C, neutral pH, and aqueous buffers, to preserve the integrity and functionality of biomolecules. Furthermore, the presence of charged amino acids, including negatively charged acidic residues and positively charged basic residues, can affect the bioconjugation process, so controlling the pH conditions is an essential requirement, and conjugation strategies must be tailored to the specific peptide sequence [58,59]. Currently, no single method for peptide conjugation is universally accepted, as each technique presents distinct advantages and limitations. Key factors to consider include the selectivity of the binding site, reaction efficiency, accessibility of reactants, and reaction rate. Among the most common methods are amine-reactive, thiol-reactive, and click chemistry reactions, which offer high levels of accessibility and selectivity [60,61].

Peptides are particularly suitable for bioconjugation due to their protein-like functionality and relative ease of their synthesis including specific amino acid sequences. Attaching peptides to the structural components of synthetic and natural materials is a common strategy to confer bioactive properties for clinical applications [62]. Peptide-biomaterial conjugates can perform various functions, such as mimicking the native extracellular matrix (ECM) and enhancing cell adhesion with application in tissue regeneration. Peptides can also provide degradable linkers for scaffold remodelling or modulate cell signalling to promote tissue growth and differentiation. 

## 5. Sheets of Peptidomimetics

Natural and synthetic peptides, including peptoids and other peptidomimetics, are gaining significant attention in materials science, nanotechnology, and biomedical engineering for their stable and intriguing self-assembled structures. Inspired by natural proteins, these peptides emulate the functions and structures of proteins like silk, ECM, fibronectin, and collagen, with potential applications in biotechnology and medicine. Leveraging their biological qualifications including biocompatibility, bioactivity, and microporous structure, peptidomimetic molecules show promise in various fields, including drug delivery, optical waveguides, photothermal conversions, and photocatalysis.

The Zuckermann group at Lawrence Berkeley National Laboratory has probed the physical properties of peptoid monolayers influencing nanosheet collapse by employing interfacial dilatational rheology. Peptoid analogues were compared to assess their nanosheet-forming abilities. Using small sample volumes, rapid and convenient rheological studies provided insights into molecular-level processes crucial for designing nanosheets with advanced functionality. Understanding the self-assembly mechanism and its tolerance to chemical modifications is crucial for designing nanosheets with advanced chemical functionality. While monolayer adsorption at the air–water interface is necessary for nanosheet formation, it is not the sole limiting factor. A set of design rules for peptoid sequences forming collapse-competent monolayers was established by characterising peptoid monolayers using surface dilatational rheology. Monolayer fluidity, represented by the residence time (τ_D_), correlates with the ability to collapse into bilayer nanosheets. Nanosheet-forming monolayers exhibit solid-like behaviour with strong interchain interactions (τ_D_ > 5000 s), while non-forming monolayers are fluid-like with weaker interactions (τ_D_ < 500 s). Small chemical modifications to peptoid chains directly impacts rheological behaviour, offering insights for sequence design and rapid screening for nanosheet-forming ability. This understanding enhances the potential applications of peptoid nanosheets in diverse fields. Zuckermann introduces an innovative approach centred on the design and synthesis of antibody-mimetic materials utilising functionalised peptoid nanosheets. While antibodies hold promise for molecular recognition in chemical and biological sensors due to their high specificity and affinity for various analytes, challenges such as poor stability and high production costs limit their widespread use in sensing devices. In addressing these challenges, this group presents a novel strategy involving the utilisation of peptoid nanosheets. These nanosheets feature a high density of conformationally constrained peptide and peptoid loops on their free-floating surfaces, creating a chemically and biologically stable extended multivalent two-dimensional material. Serving as a robust scaffold with a large surface area, the nanosheet facilitates the presentation of diverse functional loop sequences [28]. Characterisation methods such as atomic force microscopy, X-ray diffraction, and X-ray reflectivity measurements have highlighted the potential of functionalised nanosheets. These nanosheets are particularly effective as substrates for enzymes like protease and casein kinase II and templates for the controlled growth of specific inorganic materials, such as gold. This novel approach shows promise in addressing the stability and cost issues commonly encountered with traditional antibodies in sensing applications.

## 6. Application of Peptide Materials to Prevent Biofilm-Associated Infections

The widespread and easily accessible use of antibiotics for both humans and animals has led to their overuse, which in turn has driven the development of bacterial resistance. This represents a significant challenge for society, impacting both healthcare and the economy. In fact, many diseases caused by Gram-positive and Gram-negative bacteria that were once considered manageable with antibiotics are now resistant to these treatments. The current prevalence of resistant organisms is unprecedented, with many clinically important bacteria exhibiting multiple drug resistance (MDR) mechanisms [63]. Bacterial resistance mechanisms are diverse and include the enzymatic inactivation or modification of antibiotics, protection or alteration of antibiotic targets, active expulsion of drugs from bacterial cells via efflux pumps, and reduced cell membrane permeability through decreased porin expression or the production of porin variants [64,65].

An additional concern is the formation of biofilms. Pathogenic bacteria rarely exist in a free-floating (planktonic) state; rather, they tend to form micro-colonies and produce biofilms to survive in hostile environments. Bacteria within biofilms are shielded from host immune responses due to the anaerobic environment and the presence of an extracellular polymeric network and a protective slime layer, which limit the access of immune cells [66]. When biofilms are mature, nutrient depletion and the accumulation of toxic byproducts can cause the outer bacterial layers to revert to a planktonic state, enabling them to spread and cause new infections throughout the body. As a result, bacterial biofilms are a major reservoir for chronic infections, contributing to up to 80% of chronic inflammatory responses [67]. Biofilms also exhibit a high level of resistance to conventional antimicrobial treatments, as these therapies are often unable to penetrate their extracellular polymeric layer.

In natural environments, nearly all bacterial species possess the capability to form biofilms. These organised structures protect bacteria from both host immune responses and antibiotics, making infections exceedingly difficult to eradicate [21].

The use of surgically implanted medical devices has significantly increased over the past five decades, greatly enhancing the quality of life for millions of patients worldwide. Progress in biomedical research has led to the development and improvement in various medical devices, such as stents, catheters, and pacemakers. However, introducing foreign materials into the body carries an inherent risk of microbial colonisation and infection. Infections can occur during surgery or post-operatively, often originating from the patient’s own microbiota [19].

Infections associated with implanted medical devices pose a particular challenge due to the formation of bacterial biofilms. In natural environments, nearly all bacterial species have the capacity to form biofilms. Biofilms are organised systems that protect bacteria from host immune responses and antibiotics, making infections difficult to eradicate [21].

Biofilm-related infections on implanted devices often lead to device failure, requiring high doses of antibiotics and subsequent removal and replacement of the infected device. This procedure subjects patients to additional surgeries, which carry significant risks and can increase mortality. Current antibiotic treatments for device-associated infections are both expensive and often ineffective, largely due to the emergence of antibiotic-resistant bacterial strains and the risk of reinfection in a new part of the body [20,68]. 

Significant efforts are currently focused on identifying novel molecules with distinct mechanisms of action to replace antibiotics and prevent a regression to a pre-antibiotic era. Antimicrobial peptides (AMPs) represent a promising class of drug candidates capable of overcoming pathogen resistance, positioning them as strong candidates for clinical application [69].

**Figure 2:**
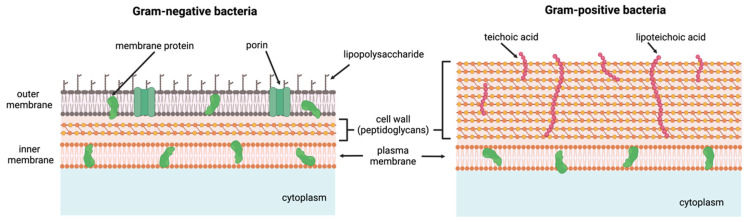
The structure of bacterial membranes differs notably between Gram-positive and Gram-negative bacteria. The primary distinction is that Gram-negative bacteria have an additional outer membrane that is rich in lipopolysaccharides (LPS). This outer membrane, located outside the thin peptidoglycan layer, acts as a barrier. In contrast, Gram-positive bacteria lack this outer membrane. Instead, they have a much thicker peptidoglycan layer that provides structural integrity and protection. This thick layer is enriched with teichoic and lipoteichoic acids, which help anchor the cell wall to the cell membrane. Created with Biorender.com.

AMPs are peptides that effectively target pathogens and are part of the innate immune system, exhibiting potent activity against a broad range of bacteria, both Gram-positive and Gram-negative bacteria that show different structural features (Figure 2). Their primary mechanism of action relies on interactions with bacterial membranes, leading to membrane disruption. To achieve this, AMPs must possess both a positive charge to facilitate electrostatic interactions with the negatively charged bacterial membranes and a hydrophobic component that allows them to insert into the lipid bilayer and cause membrane rupture. These two properties—positive charge and hydrophobicity—must be carefully balanced to ensure amphiphilicity, which is essential for their function. The membrane-targeting mechanism of AMPs also makes it more challenging for bacteria to develop resistance. Additionally, AMPs can interact with and disrupt intracellular targets, further complicating the development of bacterial resistance [70].

However, due to their broad-spectrum activity, AMPs can also pose risks, including toxicity to eukaryotic cells and disruption of the microbiota, potentially creating a niche for opportunistic pathogens. Consequently, many AMPs undergoing clinical trials have been designed primarily for topical use rather than systemic administration [71]. To mitigate some of these challenges, AMPs have been immobilised on surfaces, such as those of medical implants, to reduce biofilm formation. When tethered to surfaces, AMPs are required in lower amounts, are locally concentrated, and present minimal systemic toxicity [72]. This approach may also enhance their stability by reducing susceptibility to protease degradation. As such, AMPs offer an innovative approach to managing bacterial infections [25]. A deeper understanding of the structure–function relationships of AMPs could allow for the rational design and modification of natural AMPs, expanding the repertoire of effective antimicrobial molecules. 

### 6.1. Antimicrobial Peptide-Based Materials

An alternative approach to protecting implant surfaces is the development of antimicrobial biomaterials. Rather than directly modifying implant surfaces with AMPs, biomaterials with antimicrobial properties could be applied as coatings [73,74,75]. One method for creating these biomaterials is through self-assembly, a process widely utilized in nature for various functions. Leveraging supramolecular structures with antimicrobial activity, generated through the self-assembly or co-assembly of AMPs, represents a promising and innovative strategy [2]. Self-assembly is the spontaneous organisation of molecules into ordered supramolecular structures through non-covalent interactions without external guidance. These structures represent a state of minimum energy, with environmental forces driving the system toward thermodynamic equilibrium. Adjusting environmental conditions can shift the system to a new equilibrium, yielding different ordered structures [76].

Due to the reliance on non-covalent interactions, self-assembly is typically reversible and highly sensitive to environmental changes, allowing the modulation of activity through controlled association and the dissociation of monomers. Self-assembly enhances the protease resistance of AMPs, generating biomaterials with greater antimicrobial efficacy without increasing bacterial resistance. Studies suggest that self-assembled peptides increase amino acid side chain density, forming a spatial barrier that protects cleavage sites and reduces protease susceptibility [77]. A peptide’s ability to self-assemble depends largely on its amphiphilicity and secondary structure, which promote intermolecular interactions. Environmental factors such as pH and ionic strength also play a crucial role in controlling peptide assembly and disassembly on demand.

Specific amphipathic AMPs can create supramolecular hydrogels, with self-assembly rates controlled by pH adjustments. For example, Azoulay et al. developed self-assembling AMP hydrogels using the FKF peptide sequence [78]. This amphipathic cationic peptide formed hydrogels when dissolved in an acidic buffer, with self-assembly occurring within minutes. The resulting hydrogels exhibited β-sheet structures stabilised by hydrogen bonding and π–π stacking interactions within a pH range of 3.3 to 4.3. In vitro antibacterial assays demonstrated significant activity against *E. coli*, *P. aeruginosa*, *Acinetobacter baumannii*, and *S. epidermidis*. In vivo, the hydrogel dressing reduced bacterial presence in a rat wound model inoculated with *P. aeruginosa.* However, a 50% reduction in bacteria was accompanied by delayed wound closure, likely due to the acidic conditions necessary for hydrogel formation.

Amphipathic AMPs that self-assemble at physiological pHs can address biocompatibility concerns related to acidic aggregation conditions. D-amino acids, known for their resistance to bacterial proteases and inherent antibacterial properties, can be used to enhance the stability and efficacy of peptide hydrogels. The peptide KLVFFAK (KK-11) contains a self-assembling motif, and Guo et al. synthesised its D-amino acid analogue (KKd-11) to evaluate its antimicrobial potential. KKd-11 hydrogels formed rapidly at room temperature and effectively inhibited biofilm formation and eradicated bacteria within established biofilms of *E. coli* and *S. aureus*. Given their antimicrobial efficacy, biocompatibility, and resistance to proteolytic degradation, KKd-11 hydrogels show great potential for preventing wound infections and bacterial colonisation on medical devices [79].

Cao and colleagues created a self-assembling hydrogel using the hexapeptide PAF26 (Ac-RKKWFW-NH_2_), which aggregates at physiological pHs. PAF26 permeabilises through the microbial cell wall, leading to cell death. This amphipathic peptide forms hydrogels upon a gradual increase in pH from acid values to 7.5, with β-sheet structures confirmed in the final gel. The antimicrobial activity of PAF26 hydrogels was assessed by co-culturing them with fungi and bacteria, demonstrating a 100% killing efficiency against *Candida albicans*, *S. aureus*, and *E. coli*. While these hydrogels show promise for clinical applications, further optimisation is needed to reduce potential cytotoxicity and ensure product safety [80].

However, self-assembly in aqueous solutions is uncommon for cationic AMPs due to their positive charges. Strategies to encourage self-assembly in these peptides include chemical modifications that introduce moieties providing the necessary driving force for nanostructure formation. These modifications can involve protected amino acids, short or extended peptide sequences, lipids, or alkyl chains. 

Lipid conjugation provides hydrophobic forces that drive peptide self-assembly into various nanostructures, such as fibrils, micelles, or vesicles. This modification also enhances antimicrobial activity by increasing the peptide’s affinity for bacterial membranes, leading to membrane disruption and bacterial cell lysis [22,81].

Chemical modifications further enhance AMP stability and therapeutic potential, addressing traditional limitations by allowing the creation of antimicrobial nanomaterials with improved stability, efficacy, and controlled release properties. Examples include DP7 modified with cholesterol, which forms nanomicelles in aqueous environments [82], and human alpha-defensin 5 (HD5), modified with myristic acid, which assembles into nanospherical structures [83].

The DP7-C micelles exhibited reduced haemolytic activity compared to their unconjugated counterparts when tested on human red blood cells. They also demonstrated a maximum tolerated dose of 80 mg/kg body weight in mice following intravenous administration, highlighting their enhanced safety profile. Furthermore, the DP7-C micelles induced specific immunomodulatory responses in immune cells, leading to distinct therapeutic effects in both zebrafish and mouse models of infection. These findings position DP7-C micelles as a promising candidate for clinical applications in the treatment of bacterial infections [82].

Compared to its parent peptide HD5, the C-terminal myristoylated variant (HD5-myr) exhibits markedly enhanced bactericidal activity across a broad spectrum in vitro. The bactericidal action of HD5-myr is selective, effectively targeting *E. coli* and methicillin-resistant Staphylococcus aureus (MRSA) by disrupting their cell wall and/or membrane structures. In vivo, HD5-myr demonstrates minimal haemolytic activity and shows significantly low toxicity, indicating its potential as a safe and potent nanobiotic agent for antimicrobial applications [83].

Modifications to peptides can include the addition of linkers, as demonstrated in a recent study where a three-arginine-proline (RP) repeating peptide was modified with flexible glycine linkers (GGG) and hexadecanoic acid (C16) to create C_16_-3RP dendron nanoparticles [84]. These nanoparticles exhibited robust antimicrobial efficacy and stability. In animal models, C_16_-3RP nanoparticles significantly reduced bacterial loads across multiple organs, including the liver, spleen, lungs, and kidneys, and were shown to lower serum pro-inflammatory cytokine levels. Analysis using biofluorescence, microscopy, and transcriptome sequencing revealed that C_16_-3RP nanoparticles eliminate Gram-negative bacteria through multiple mechanisms. These include increasing membrane permeability, causing cytoplasmic membrane depolarisation and severe membrane disruption, inhibiting ribosome biogenesis, and affecting energy generation and other cellular processes. The therapeutic benefits of C_16_-3RP treatment extended to reducing or preventing tissue damage, as evidenced by significantly fewer histological abnormalities in treated versus control tissue samples. Importantly, the nanoparticles showed promising biocompatibility and in vivo efficacy without apparent resistance development, making them a promising therapeutic option for bacterial infections [84].

In a different approach, Adak et al. developed a lipopeptide-based hydrogel by conjugating palmitic acid (C16) with the peptide sequence NAVSIQKKK (PA-NV). This structure leverages the amphiphilic properties of PA-NV, with NAVSIQ acting as a crucial intermediary segment between C16 and the cationic KKK motif. The amphiphilic character drives self-assembly into β-sheet structures, which facilitate hydrogel formation. These PA-NV hydrogels display strong biocompatibility, showing resistance to enzymatic degradation (specifically to proteinase K) and low cytotoxicity in mammalian cell assays. Furthermore, human red blood cells (RBCs) largely retained their native morphology after exposure, indicating minimal haemolytic activity. The cationic and amphiphilic nature of PA-NV also enables effective interactions with bacterial cell membranes, enhancing membrane disruption and bacterial eradication. This balance of stability, safety, and antimicrobial activity positions PA-NV hydrogels as promising candidates for clinical applications, with further in vivo studies needed to fully explore their therapeutic potential [85].

Another modification of AMPs to achieve self-assembling peptides involving multiple alterations (both electrostatic and hydrophobic forces) was proposed by Lombardi et al. who engineered the antimicrobial peptide WMR to enhance its antibacterial and antibiofilm activities, particularly against the Gram-negative bacterium *Pseudomonas aeruginosa* and the fungus *Candida albicans* [86]. 

**Figure 3:**
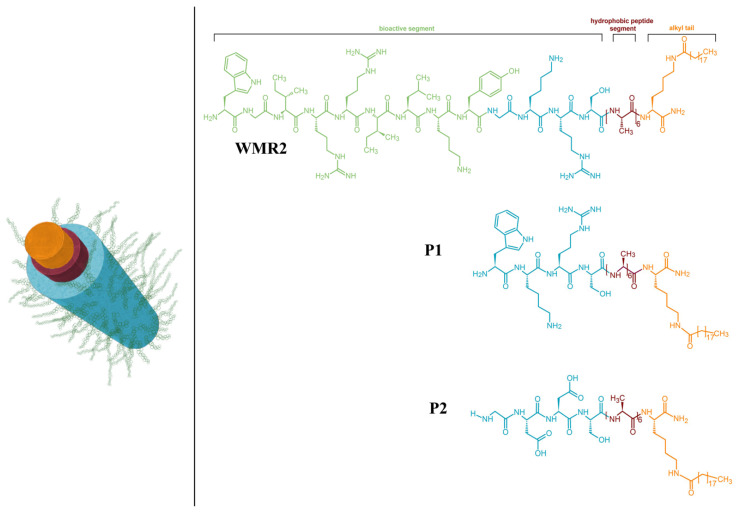
Molecular structures of WMR2PA, PA1, and PA2 and their suggested self-assembled nanostructure. “Reproduced with permission from ref. [87]. Copyright 2019, American Chemical Society”.

WMR was modified at the C-terminus by adding a sequence of six alanines and a hydrophobic 16-carbon tail, creating the WMRPA molecule. By modifying WMR at the C-terminus with a six-alanine sequence and a hydrophobic 16-carbon tail, they created the amphiphilic peptide WMRPA (Figure 3). This modified molecule was self-assembled into fibres, with the alanine sequence and C16 tail forming the core and the WMR peptide exposed on the fibre surface [87]. These fibres effectively eradicated biofilms and inhibited their formation at low concentrations, down to 20 μM. The study also showed that WMRPA could co-assemble with shorter oppositely charged peptides such as GDDS (PA2) and WKRS (PA1). This co-assembly allowed the precise modulation of AMP concentration on the fibre surface, enhancing WMR’s antimicrobial activity within the assembly and enabling further reduction in AMP concentrations to minimise toxicity while retaining biofilm eradication effectiveness [87]. Additionally, this strategy facilitates the integration of multiple components, including AMPs and conventional antibiotics, offering a promising approach for reducing antibiotic doses and mitigating resistance development.

In addition to multichain structures made of multiple peptides that self-assemble, single-chain organisation is also significant for biological function. Recent advances in the design of single-chain polymer nanoparticles (SCNPs) aim to replicate the sophisticated folding observed in proteins. SCNPs, synthetic polymers with intrachain folding driven by crosslinking or noncovalent interactions, exhibit protein-mimetic structures. While SCNPs may lack the precision of folded proteins, their morphology resembles that of intrinsically disordered proteins (IDPs). Unlike well-defined proteins, IDPs fold on a complex energy landscape with transiently coexisting structures. SCNPs, with biomimetic functions like enzyme-like catalysis, demonstrate their potential to mimic and expand upon the roles of natural biomaterials, showcasing their importance in synthetic biology [88].

Beyond modifications with amino acids or lipid groups, other materials—such as polymers, ceramics, metals, and composites—have been incorporated to create AMP-based hybrid materials. These advanced materials exhibit microbicidal activity against single or multiple pathogens, expanding the potential applications of AMPs in antimicrobial therapies.

Polymeric hydrogels have garnered significant attention in defeating resistant infections. Hydrogels are soft biomaterials composed of highly hydrated polymeric networks, which can be engineered with various functional groups for applications in antibacterial therapy, tissue regeneration, and drug delivery [89]. Due to their customisable nature, hydrogels can physically incorporate or tether a wide range of antimicrobial agents, offering a versatile platform to combat biofilm-associated infections [86,87,90].

The first is the simplest approach and involves directly incorporating AMPs into polymer blends, which maintains their functional efficacy without requiring complex preparation steps. For example, the antimicrobial peptide Tet213 was directly added to a collagen solution following direct mixing with no reported decline in its functional activity [91]. Similarly, the AMP piscidin-1 was introduced into chitosan scaffolds after scaffold fabrication [92].

Cross-linkers can also be utilised to attach AMPs to polymers. For instance, extracellular matrix-derived peptides were added to a chitosan matrix using the cross-linker maleimidobenzoyl [93].

The second approach is about the conjugation of AMPs to the surface of polymer networks and primarily aims to enhance their stability and reduce toxicity while preserving their antimicrobial efficacy. Thus, in place of entrapping the peptides within the polymeric network, AMPs are immobilised onto solid surfaces. This can be accomplished through methods like simple adsorption, electrostatic interactions, or more stable covalent bonding. While physical adsorption can increase peptide stability, it presents challenges in controlling the degree of immobilisation and the release rate, as these processes rely on passive diffusion. Covalent conjugation offers more precise control over peptide concentration and retention time at the target site, thereby minimising side effects and reducing accumulation in organs such as the brain, liver, and spleen. This method typically involves modifying both the AMP and the polymer with functional groups necessary for chemoselective conjugation reactions. AMPs can be functionalised without significantly compromising their antimicrobial activity [94]. Cathelicidin LL-37 was conjugated onto a collagen scaffold by reacting the peptide on hydrophobic PDMS mats [95]. Additionally, it was observed that AMPs immobilised on the surface demonstrated higher antimicrobial activity than those that were encapsulated in the core of the hydrogels. However, this difference might be attributed to the availability of the peptides themselves rather than the encapsulation method.

Antimicrobial peptides can be conjugated to polymers via their N- or C-terminus. Since one of the termini is often more critical for antimicrobial activity, conjugation should be carried out via the terminus that is less involved in this activity. For example, anoplin that was conjugated to chitosan at the C-terminus was more effective against certain bacteria than N-terminal conjugation [96]. Similar results were observed with Dhvar5 conjugated to chitosan, where N-terminal conjugation provided stronger anti-adhesive effects, while C-terminal conjugation showed higher bactericidal activity [97,98]. Conjugation through amino acid side chains is usually avoided due to the potential loss of antimicrobial activity. Generally, hydrophobic amino acids should be positioned away from the linkage site to enhance antimicrobial activity. However, this is not a universal rule. 

Another key factor is the density of tethered AMPs on the surface with a minimum effective concentration required to achieve bacterial killing [23]. A direct correlation exists between peptide concentration and antimicrobial activity, as higher graft densities often force peptides to interact with bacterial membranes. However, some bacteria, like *S. aureus*, can become insensitive to increased AMP surface density. The choice of polymer plays a crucial role in enhancing AMP characteristics. Hydrophilic polymers, whether neutral or cationic, minimise the formation of a protein corona around the AMP–polymer conjugate, which can otherwise reduce antimicrobial activity by blocking interactions with bacteria. Neutral hydrophilic polymers increase solubility and lower cytotoxicity to mammalian cells, while cationic polymers, despite increasing potency, may cause higher toxicity [24]. Attention should be also paid to the polymers’ molecular weight. Higher molecular weight polymers may hinder access to bacterial membranes due to steric hindrance, reducing their likelihood of promoting membrane destabilization [24].

Antimicrobial peptides, being macromolecules, can have significant and varying impacts on the scaffolds into which they are incorporated, depending on the specific AMP used. For instance, the incorporation of AMP Tet213 into an alginate/hyaluronic acid/collagen wound dressing scaffold was found to negatively affect porosity. Although the porosity decreased, it remained close to the optimal porosity required for wound dressing applications. This reduction in porosity also contributed to a lower swelling rate. However, Tet213 had a slightly positive effect on the tensile strength of the scaffold, enhancing its mechanical stability in the presence of lysozyme [91]. Other AMPs, such as piscidin-1, have been shown to produce highly porous chitosan scaffolds with favourable biodegradation rates [92]. On the other hand, cathelicidin LL-37 exhibited strong antibacterial activity against a range of pathogens, including *Escherichia coli*, *Pseudomonas aeruginosa*, *Staphylococcus aureus*, methicillin-resistant *Staphylococcus aureus* (MRSA), and *Bacillus subtilis*. Cathelicidin was successfully bound to the Puracol skin model, demonstrating improved retention compared to the unmodified sequence [99]. 

Conjugation of AMPs to nanoparticles (NPs) is gaining interest due to their physicochemical properties, such as size and shape. NPs allow for higher AMP loading and require lower doses than soluble AMPs [100,101]. AMP-NP conjugates can more effectively penetrate bacterial biofilms, as their size, shape, surface charge, and composition facilitate initial interactions and penetration [102]. However, AMP conjugation to NPs often diminishes antimicrobial activity, potentially due to aggregation and surface effects that alter AMP secondary structures [24]. This issue can be mitigated by introducing large linkers like PEG, which provide conformational freedom, improving the AMP–bacteria interaction [103].

### 6.2. Mechanism of Action of Antimicrobial Biomaterials

Over the past few decades, research has largely categorised the mechanisms of AMPs into two primary types: those that target bacterial membranes and those that disrupt intracellular processes. Numerous models explain how AMPs interact with bacterial membranes. The most common mechanisms involve the formation of pores in the bacterial lipid membrane [15], though some mechanisms disrupt lipids without creating pores, such as membrane thinning [16] or thickening [104].

**Figure 4:**
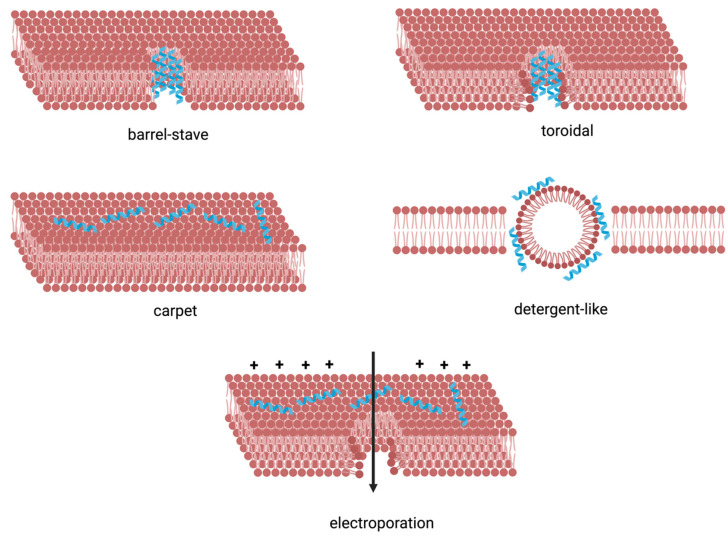
The primary models explaining pore formation in bacterial membranes when interacting with AMPs. They include the barrel-stave model, toroidal-pore model, carpet model, detergent-like model, and electroporation model. Created with Biorender.com.

The primary models explaining pore formation by AMPs include the barrel-stave model, toroidal-pore model, carpet model, detergent-like model, and electroporation model (Figure 4).

In the barrel-stave model, the AMP adopts an amphiphilic structure—usually α-helices—upon interacting with the phospholipid bilayer and inserts perpendicularly into the membrane. This interaction creates a gap in the membrane’s hydrocarbon tail region, inducing positive curvature and thinning the membrane. The AMP then embeds vertically, forming a compact pore with hydrophobic residues aligning with the bilayer’s core. Some peptides, such as those containing unnatural amino acids like aminoisobutyric acid (Aib), ceratoxins, and alamethicin, use this mechanism [105,106].

The toroidal-pore model involves interactions between the polar residues of the AMP and the phospholipid head groups, facilitating the rapid passage of ions, small molecules, and even phospholipids as they flip-flop across the membrane. Part of the peptide may also integrate into the bilayer. Magainin and melittin, well-known AMPs, exemplify this mechanism [107].

Peptides like cecropin form a “carpet” on the membrane’s surface at high peptide-to-lipid ratios, especially with negatively charged phospholipids. This disrupts the membrane’s stability, and the accumulation of AMPs reduces electrostatic repulsion, ultimately causing membrane lysis. These same AMPs act like detergents at lower concentrations, forming micelles with the lipid bilayer. When the concentration surpasses their critical micelle concentration (CMC), AMPs aggregate and destabilise the membrane by dissolving the bilayer, collapsing the electrochemical gradient, and releasing cytoplasmic contents [108].

The electroporation model applies to highly charged AMPs that can generate an electrostatic potential across the bilayer. If this potential reaches about 0.2 V, transient pores are formed without structural changes to the membrane. These pores, with diameters of 2–4 nm, allow small molecules to pass through and may facilitate AMP entry into the cell, thus inhibiting intracellular processes [15].

Beyond disrupting membranes, some AMPs target intracellular functions by interfering with essential bacterial processes, such as peptidoglycan synthesis, DNA replication, protein synthesis, and the activation of the SOS response. For example, AMPs that inhibit peptidoglycan synthesis can thin the peptidoglycan layer from 30–34 nm to 17–20 nm, weakening the cell wall and leading to osmotic lysis. Other AMPs enter the cell and block DNA replication or protein synthesis, resulting in bacterial cell death [109].

This dual functionality—targeting both the membrane and intracellular processes—makes AMPs promising candidates for novel antimicrobial strategies.

The mode of action of AMPs changes when integrated into supramolecular structures. In this case, peptides are immobilised within the biomaterial, limiting their conformational flexibility and restricting their ability to form membrane pores or interact with intracellular processes [61]. The biomaterial matrix, ranging in size from nanometres to micrometres, effectively immobilises peptides, altering their mechanism of action [110].

**Figure 5:**
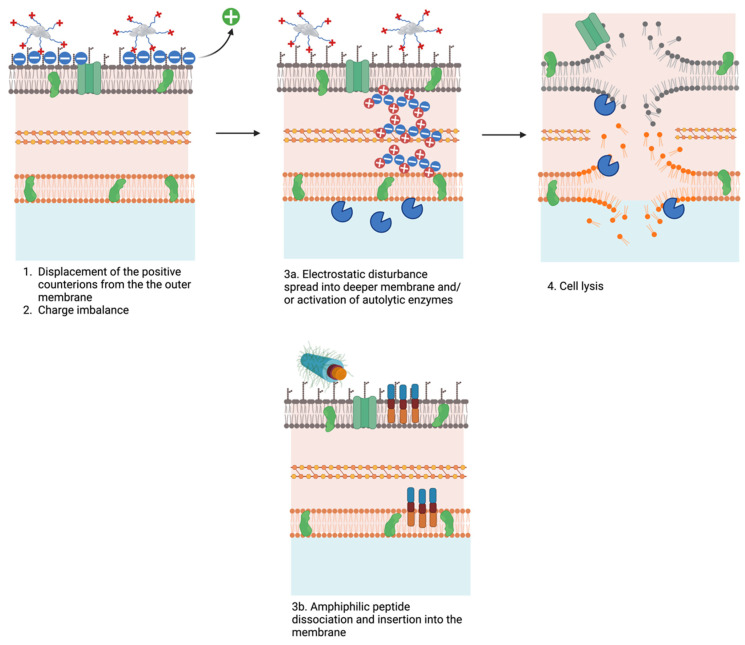
Proposed mechanism of action for AMP biomaterials on a Gram-negative bacterium. Initially, the positively charged AMP interacts electrostatically with the negatively charged bacterial membrane, resulting in a localised high concentration of AMP. This interaction disrupts the membrane’s electrostatic balance, impacting inner membrane layers and altering membrane potential. Subsequently, autolytic enzymes are activated, initiating a cascade of degradation events. These effects culminate in cell death, characterised by membrane lysis. Additionally, in self-assembling materials, it has been proposed that individual peptide monomers can detach from the material and interact directly with the lipid membrane, a process that is particularly facilitated when the AMP is conjugated to a lipid tail. Created with Biorender.com.

With biomaterials, antimicrobial activity occurs through direct contact with biofilms or isolated microbes. When the biomaterial interacts with bacterial cells, it “wraps” or “traps” the bacteria, isolating them from the environment and blocking nutrient flow, which inhibits cell proliferation [111]. Moreover, the adhesion of the antimicrobial biomaterial to the biofilm network induces membrane stress, disrupting the organisation of phospholipids (Figure 5). The stress induced on bacterial membranes is likely driven by the increased local concentration of antimicrobial peptides (AMPs) in conjugated form. Unlike AMPs in solution, which typically need to be inserted into or accumulate on bacterial membranes to induce cell death, conjugated AMPs exert their effects without this requirement. Instead, the dense presence of positively charged amino acids within the AMP sequence generates a strong attraction to the negatively charged bacterial membrane surface. This intense electrostatic interaction displaces positively charged counterions from the bacterial outer surface, disrupting the membrane’s electrostatic stability [23,112]. The resulting imbalance alters the membrane potential, potentially triggering lethal processes such as the activation of autolytic enzymes or the spread of this electrostatic disturbance into deeper membrane layers. Furthermore, this disruption of charge balance can lead to osmotic imbalances, causing water influx and leading to swelling and potential cell lysis [113].

In self-assembling peptide structures, individual monomers may separate from the aggregate and integrate into bacterial membranes. This mechanism is evident in the case of the WMRPA peptide, where research highlights the critical role of the lipid tail in biofilm eradication. While the lipid tail primarily supports the self-assembly process by stabilising the core structure, the study shows that it also contributes directly to antimicrobial activity. This suggests that some monomers can dissociate from the aggregate and function independently, enhancing the bactericidal effects in synergy with the biomaterial [87]. When these monomers penetrate bacterial membranes, they compromise the structural integrity of the lipid bilayer, facilitating membrane disruption and, ultimately, cell death. This combined mechanism allows the self-assembling peptide structures to act both as a collective unit and through detached monomers, which interact independently with bacterial cells to potentiate antimicrobial action. Further penetration compromises the bilayer structure, eventually causing rupture and releasing cytoplasmic contents, including DNA, RNA, and proteins.

Peptide materials with membrane-directed antibacterial activity have also been shown to disrupt bacterial respiration. This interaction interferes with membrane-bound respiratory dehydrogenase enzymes essential for the bacterial respiratory chain. These enzymes depend on their orientation and association with the lipid bilayer, and physical disruption can displace these proteins, leading to loss of structure and inactivation. As a result, bacterial respiration is uncoupled from oxidative phosphorylation, causing metabolic arrest and, ultimately, bacterial cell death [110]. 

This effect is particularly pronounced in Gram-positive bacteria, where respiratory enzymes are localised in the single-cell membrane. In contrast, Gram-negative bacteria possess two membranes, with their respiratory enzymes embedded in the inner membrane [114]. This double membrane provides additional resistance to antimicrobial agents. However, biomaterials incorporating AMPs with a lipid tail can penetrate the outer membrane and eliminate even established biofilms in Gram-negative bacteria, such as *Pseudomonas aeruginosa* [87]. 

## 7. Application of Peptide Materials for Drug Delivery in Cancer 

An effective drug delivery system should demonstrate high drug-loading capacity, minimal leakage during transport, targeted delivery to diseased tissues, and controlled release of the therapeutic compound. Due to their biocompatibility, biodegradability, and multifunctionality, self-assembled peptides serve as excellent carriers for transporting small molecules, protein-based therapeutics, cytokines, and genetic material. This delivery strategy can significantly enhance the solubility of poorly soluble drugs, increase the specificity of active agents, and prolong the therapeutic effect [115,116]. Peptides are widely used in drug delivery and oncology applications because of their high selectivity, low toxicity, and ease of chemical modification.

The EGF-EGFR signalling pathway is a critical target for developing selective chemotherapeutic interventions. As a result, two primary types of EGFR inhibitors have been approved for clinical use: monoclonal antibodies and small molecule kinase inhibitors. Developing effective drug and gene delivery vectors is crucial for improving cancer therapies. To this end, a peptide amphiphile (EGFR-PA) was engineered to bind specifically to the epidermal growth factor receptor, forming the main framework for creating ultra-stable self-assembling peptide nanovesicles (EGFR-SPV) [117]. The synthesised EGFR-SPV demonstrated efficient encapsulation of various therapeutic agents, such as drugs, siRNAs, and quantum dots, and showed strong affinity and targeting specificity towards EGFR-expressing cancer cells. Compared to cationic liposomes, EGFR-SPV exhibited superior delivery capabilities, achieving significantly higher drug or plasmid DNA delivery to tumour sites and boosting gene expression by a factor of three. Additionally, this delivery system proved highly effective for co-delivery. The combined delivery of doxorubicin and the acetylcholinesterase gene resulted in enhanced drug and gene delivery in both in vitro and in vivo experiments. This led to a notable suppression of tumour growth in a liver cancer xenograft model [117]. 

Small molecules like the drug taxol have shown the ability to self-assemble into nanostructures, effectively delivering taxol and other hydrophobic anticancer drugs. A cell-penetrating peptide (CPP) was conjugated to taxol to enhance cellular uptake, resulting in a novel compound (taxol-CPP). This new material was characterised and tested on HepG2 liver cancer cells. The resulting nanospheres, approximately 130 nm in size, retained the therapeutic efficacy of taxol. Furthermore, these nanospheres could co-deliver doxorubicin, offering a more potent combined anticancer effect [118]. 

Nanotubes are highly efficient in encapsulating drugs, enabling targeted delivery to specific cellular organelles. For instance, Fmoc-diphenylalanine (Fmoc-FF) and its derivatives self-assemble into various nanostructures that promote sustained drug release, particularly under acidic conditions. Fmoc-FF-based hydrogels can selectively trap larger fluorescent molecules, such as FITC-labelled insulin. In contrast, smaller fluorescent compounds like fluorescein are confined within the hollow cores of the nanoparticles, facilitating controlled and gradual drug release [119]. The gradual addition of Fmoc-FF aqueous solution into mineral oil containing vitamin E polyethylene glycol succinate (E-TPGS), which serves as both an emulsion stabiliser and a source of hydroxyl functional groups, led to the self-assembly of dipeptides into hydrogel nanoparticle aggregates. The size of these aggregates could be finely adjusted by optimising the emulsion process parameters, including stirring speed and the solvent volume ratio [120]. 

Furthermore, these hydrogels’ delivery efficiency and selectivity can be tailored for different drugs. For example, when encapsulating doxorubicin (Dox) and 5-fluorouracil (5-Fu)—two drugs with distinct chemical structures, molecular weights, and hydrophobicities—within hydrogel nanoparticles, their release profiles exhibit significant differences. In the case of 5-Fu, 50% of the drug was released within 5 h, with the release rate plateauing after 12 h. In contrast, Dox showed a slower release, with 50% released after 20 h and 80% released after 55 h, without reaching a plateau. This prolonged release is attributed to the higher molecular weight of Dox and the extended aromatic interactions between Fmoc-FF and Dox molecules [119,120].

Xu et al. synthesised a novel peptide analogue, Fc-FFRGD, composed of a ferrocene (Fc) group, an FF dipeptide, and an RGD motif. Due to its amphiphilic properties and strong non-covalent interactions, this compound could form stable nanostructures and hydrogels in an aqueous environment. Upon self-assembly in water at neutral pH, the molecule initially formed metastable spherical aggregates, which then transformed into nanofibres over a 2-h period when aged at room temperature. Notably, the nanoscale RGD clusters on the surface of these nanostructures make them potentially useful as biomimetic matrices for cell culture and as vectors for targeted drug delivery through multivalent RGD–integrin interactions. This material demonstrates significant potential as a biomimetic scaffold for cell adhesion and growth and a nanocarrier for drug encapsulation and delivery [121]. 

Additionally, peptide nanorods and chimeric peptides have been developed for targeted and synergistic cancer therapies, offering improved cellular uptake and nuclear localisation upon light irradiation [122]. Cheng et al. engineered chimeric peptide nanorods, denoted as [C_16_-K(PpIX)]-PKKKRKV- (PEG)_8_], which incorporate both hydrophobic alkyl chains and the photosensitiser PpIX to achieve targeted delivery to both the plasma membrane and the cell nucleus [123]. The hydrophobic C_16_ alkyl chain within the peptide sequence promoted selective interaction with the plasma membrane, while the nuclear localisation signal sequence (PKKKRKV) directed translocation to the nucleus. This combination of nuclear-targeting peptide and alkyl chain enhances cellular uptake, especially by increasing membrane permeability and promoting necrotic entry pathways. Upon light activation, these positively charged self-assembling nanorods significantly increase the nuclear localisation of the therapeutic agents. Despite promising laboratory results, therapeutic peptides’ clinical use is often limited by their poor ability to penetrate cell membranes effectively. One strategy to overcome this challenge is to locally perturb the plasma membrane, allowing the biomaterial to pass through and enhancing the internalisation and anticancer efficacy of peptide drugs. Building on this approach, an innovative system was developed that leverages enzyme-specific interactions at the cell membrane interface to promote in situ self-assembly and induce phase separation of lipids and proteins on the membrane [124]. Specifically, the enzyme alkaline phosphatase (ALP), which is overexpressed on the surface of certain cancer cells, was specifically targeted in this approach. The pro-apoptotic peptide [KLAKLAK]2 (KLAK) was modified by attaching a phosphate-containing tyrosine (KYp) through the amino group of lysine. In its solution form, this modified peptide exists as a single chain. ALP on the cell surface selectively recognises the phosphorylated peptide substrate and catalyses its dephosphorylation. This enzymatic reaction exposes hydrophobic benzene rings, causing the modified peptides to aggregate significantly and form nanoparticles. The assembly process results in the clustering of ALP on the membrane, concentrating both ALP and the aggregated peptides into a larger complex. This clustering effect induces the phase separation of lipids and proteins on the membrane, increasing membrane permeability and facilitating enhanced uptake of peptide drugs into the cell [124].

This strategy enhances the efficiency of peptide drug delivery and provides a targeted approach by exploiting the overexpression of specific enzymes in cancer cells, thereby improving therapeutic outcomes.

**Figure 6:**
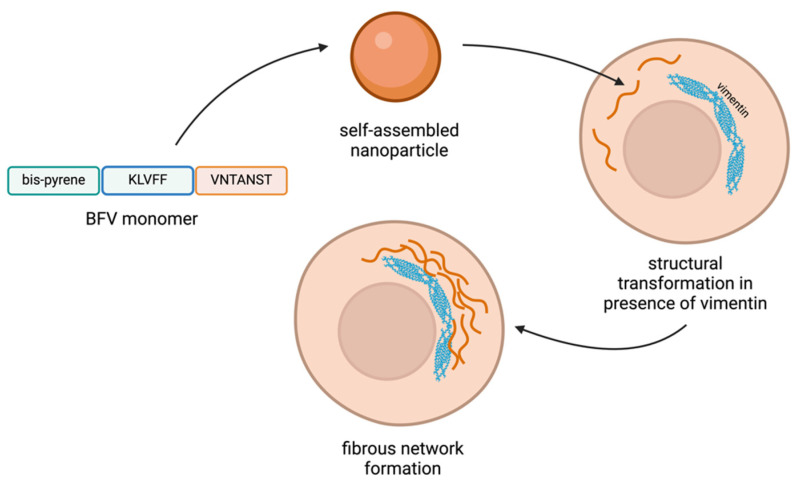
Mechanism of action of the BVF. BVF is a peptide that can form spheres but it transitions into fibres in the presence of vimentin within the cell. The fibre network then envelops vimentin, preventing the tumour from migrating to other sites. Created with Biorender.

In the case of breast cancer, the overexpression and abnormal self-assembly of vimentin protein contribute to changes in cell shape and enhanced tumour cell motility. To address this, Wang et al. developed a binding-induced fibrinogenic peptide (BFV) aimed at forming a fibre network in situ, which prevents the improper assembly of vimentin (Figure 6). The BFV peptide consists of three key functional domains: KLVFF, an assembly sequence that facilitates the formation of BFV nanofibers through hydrogen bonding; VNTANST, a targeting sequence that specifically binds to vimentin and activates the fibrogenesis process; and Bis-pyrene (BP), a fluorescent marker used to track the BFV nanoparticles. When the BFV peptide binds to vimentin, it triggers the fibrogenesis process, forming a synthetic peptide fibre network. This network disrupts vimentin’s normal polymerisation, thereby hindering tumour cell migration and invasion by destabilising their structural framework [125].

## 8. Self-Assembling Peptides as Matrix Mimetics in the Pharmaceutical Market and Clinical Trials

Several self-assembling peptides have successfully transitioned from research and development into the clinical setting, demonstrating their efficacy in the pharmaceutical market. These products are notable for their ability to improve drug delivery, enhance therapeutic efficacy, and offer more patient-friendly dosing regimens. With their 3D fibrous networks, peptide-based self-assembled materials are utilised in commercial products, such as Matrixyl in dermatological formulations and C_16_-GHK for addressing skin aging issues. PuraMatrix, another commercially available peptide gel, is used in 3D tissue cultures, promoting tissue growth with biodegradable and nonimmunogenic properties [126]. Recognising the significance of self-assembled peptide-based materials and understanding the self-assembly process and relevant techniques becomes crucial. While short peptides are economically viable and easily manufacturable, ultrashort peptides (up to seven amino acids) also exhibit self-assembly capabilities, offering the advantage of prompt sequence fine-tuning for specific requirements [127]. Ultrashort peptides have applications that extend beyond biology, including toxic metal detection, wastewater treatment, crop protection, and green energy generation. Their diverse physicochemical properties in different organic solvents make them useful for compound purification and as platforms for chemical catalysis. With their mild and rapid synthesis, along with the ability to be fine-tuned through modifications, functionalisation, and self-assembly, ultrashort peptides are versatile biomaterials suited for a wide range of applications [128].

Other examples of peptide materials on the market include the well-studied sequences lanreotide and RADA16. Lanreotide is a synthetic octapeptide analogue of somatostatin, a natural compound that typically regulates growth hormones and inhibits the secretion of other hormones such as serotonin, gastrin, and pancreatic polypeptide [129]. Due to its rapid degradation in the body, lanreotide has been explored for its self-assembly behaviours. The peptide was engineered as a more pharmacologically stable form. This compound emulates the inhibitory effects of somatostatin on hormone release, including growth hormone and insulin-like growth factor-I (IGF-I), positioning it as a key therapeutic agent in the management of acromegaly and certain gastroenteropancreatic neuroendocrine tumours (GEP-NETs) [130]. The development of lanreotide started with the sustained release (lanreotide SR) formulation using a microparticle-based drug-delivery system to enhance patient adherence, which required intramuscular injections every 7 to 14 days [131]. Later, in 2007, the FDA approved lanreotide autogel (ATG) (marketed as Somatuline^®^ Depot), an advanced formulation containing lanreotide acetate in a supersaturated aqueous solution, delivered as a deep subcutaneous injection every four weeks, significantly reducing the treatment frequency [132]. The self-assembly characteristics of lanreotide are integral to its controlled-release mechanism. Its cyclic structure, stabilised by disulphide bridges, facilitates the formation of dimers that subsequently arrange into β-sheet-rich filaments through non-covalent forces, such as hydrogen bonding and π–π interactions [133]. These filaments then form nanotubes, forming a hydrogel structure in aqueous environments. 

These hydrogels gradually disassemble, permitting consistent lanreotide serum levels with a predictable elimination half-life of approximately 30 days [134]. A recent study explored the pharmacokinetics and safety of an extended-release formulation, aiming to extend dosing intervals to 8–12 weeks for improved patient adherence [135]. The results indicated stable growth hormone and insulin-like growth factor-I (IGF-I) levels without compromising safety, confirming that serum lanreotide concentrations could be sustained at therapeutic levels with longer intervals between doses. 

Additionally, the formulation incorporates acetic acid (AcOH) as a counterion, which plays a crucial role in modulating the self-assembly behaviours of lanreotide containing two basic amino acids [132].

RADA16 is a synthetic ion-complementary self-assembling peptide designed after EAK16 and has been extensively studied for its unique properties and diverse biomedical applications. The peptide consists of alternating hydrophilic and hydrophobic amino acids with a charged distribution as + − + − + − + −, allowing it to self-assemble into β-sheet nanofibrous structures in physiological environments. This self-assembly results in the formation of hydrogels, which possess high stability and mimic natural ECM. Due to its biocompatibility, low immunogenicity, and ability to support cell attachment and proliferation, researchers and surgeons have utilised the distinctive features of RADA16 in various experimental and clinical settings [136,137]. Commercialised as PuraMatrix™ (1% RADA16), RADA16 functions as an ECM substitute for investigating cell adhesion, chemotaxis, proliferation, and development in laboratory settings, and as a 3D scaffold for in vitro and preclinical in vivo studies on wound healing, and tissue engineering [138]. 

However, the reliance of RADA16 on weak intermolecular forces, such as hydrophobic and hydrogen bonding, limits its mechanical stability in high-stress environments, such as joint or load-bearing tissues. This limitation has led researchers to explore strengthening strategies, such as incorporating RADA16 into composite hydrogels with durable polymers like poly (L-lactic-co-glycolic acid) (PLGA), which enhanced stability and supported nerve repair markers in vitro models [139]. Additionally, integrating motifs like RGD improved cell adhesion and wound healing performance in preclinical spinal cord models [140].

Another application of RADA16 is as a hemostatic agent marketed as PuraStat^®^ (2.5% RADA16), which is a CE-marked class III medical device used to control bleeding during surgeries by forming a physical barrier that stabilises clot formation [141]. 

The shear-thinning properties make it suitable for low-pressure bleeding environments but may limit its effectiveness in high-shear settings, such as intra-arterial procedures. Researchers have suggested that increasing peptide concentration or modifying the structure of RADA16 to enhance crosslinking could improve its resistance in such applications [142]. PuraStat^®^ has gained approval in several regions, including Europe and Asia, for use in gastrointestinal endoscopic procedures and surgeries to prevent delayed bleeding. It offers a safe profile with minimal side effects, making it a widely accepted solution in clinical settings. Additionally, RADA16 has found utility in wound care, marketed as PuraDerm^®^ in the United States for treating complex wounds, including diabetic and surgical ulcers [142]. Another formulation approved by the FDA in 2019, PuraSinus^®^ (2.5% RADA16), is designed to prevent adhesions following nasal surgery and promote wound healing in the sinus cavity [143].

## 9. Conclusions and Future Vision

Peptides have great potential in the development of advanced biomaterials, owing to their unique properties such as self-assembly, responsiveness to biological stimuli, and selective binding to specific tissues or cells. These characteristics position peptides to drive significant advances in various fields, including tissue engineering, drug delivery systems, and medical implant coatings that promote integration while minimizing the risk of infection. As our understanding of disease pathways continues to deepen, peptides can be further modified to carry small molecules or receptor domains, improving therapeutic outcomes. Customizable to respond to environmental changes within the body, these biomaterials can enable on-demand targeted drug release and dynamic adjustments to better integrate with surrounding tissues.

Synthetic peptides offer exceptional versatility in conjugation chemistry and can be engineered to replicate the active amino acid sequences of proteins, enhancing their binding affinity for specific targets. Chemical modifications, such as cyclisation and the use of peptoids, can also improve peptide stability, making them more suitable for clinical applications.

Peptides hold great promise in addressing several key medical challenges, including tissue regeneration, infection control, and targeted drug delivery. In particular, the food industry has shown interest in developing self-assembled antimicrobial peptides that prevent bacterial and fungal resistance without targeting specific molecular sites. Embedding AMPs within these self-assembling systems enhances antimicrobial activity against a broad spectrum of Gram-positive and Gram-negative bacteria, while minimising the risk of resistance. This innovative approach paves the way for advanced antibacterial materials. Combining AMPs with conventional antibiotics offers a new avenue for reducing antibiotic dosages and curbing resistance development.

Furthermore, self-assembling peptides have emerged as promising platforms for cancer drug delivery, offering advantages such as biocompatibility, tuneable structural properties, and responsiveness to biological stimuli. Recent advances demonstrate the versatility of peptides in forming nanostructures that can encapsulate various chemotherapeutics, protect them from degradation, and enable targeted delivery to tumour cells, minimising off-target effects. The multivalent presentation of functional groups on these nanostructures enhances their interactions with cancer cells, improving drug uptake and therapeutic efficacy.

While significant progress has been made, self-assembling peptides are moving towards commercialisation, with some products in development or already on the market. The ability of peptides to self-organise into nanostructures offers distinct advantages in fields ranging from drug delivery to tissue engineering and wound healing. PuraMatrix, a prime example, showcases the practical application of peptide-based biomaterials in clinical settings. However, transitioning from laboratory research to clinical use requires extensive effort and interdisciplinary collaboration among materials scientists, chemists, biologists, and clinicians.

Despite these advancements, challenges remain. Key issues include optimising peptide stability in three-dimensional self-assembled structures, particularly in hydrogels, and reducing potential cytotoxicity. Addressing immunogenicity is also an ongoing concern. Additionally, self-assembling peptides can be sensitive to environmental conditions such as temperature, pH, and ionic strength, which could affect their stability during storage, transportation, and use. For example, peptide nanocarriers can be unstable under physiological conditions, requiring precise control over environmental factors such as pH and temperature [144]. Nanovesicles and micelles may disintegrate upon dilution, reducing drug delivery reliability and therapeutic efficacy. The low critical micelle concentration (CMC) of some peptide micelles can lead to premature drug release, affecting their stability and clinical viability [145]. Ensuring stability in formulations, especially for drug delivery applications, is crucial to maintaining their structural integrity and efficacy over time.

While self-assembling peptides are generally considered biocompatible, potential toxicity, especially in long-term or high-concentration applications, remains a concern. Nanocarriers may trigger immune responses or be cleared rapidly by the body, impacting their effectiveness [36]. These challenges are particularly relevant in cancer treatment, where high-dose frequent administration may be required.

Another challenge is scaling up production for clinical use while ensuring consistent in vivo performance. The cost of producing self-assembling peptides, particularly for large-scale pharmaceutical or medical applications, could be higher than that of traditional small-molecule drugs due to peptide synthesis, purification, and formulation costs.

Nevertheless, continued innovation in self-assembling peptide systems holds great promise for next-generation cancer therapeutics and targeted drug delivery. Self-assembled AMPs show significant potential in pharmaceutical sciences and biomedical engineering despite certain challenges that need to be addressed for commercial use. However, these promising systems represent a considerable opportunity that cannot be overlooked. Addressing these challenges will be pivotal for driving further progress. As research advances, peptides are expected to play a crucial role in significantly enhancing the specificity and functionality of biomaterials, potentially transforming the landscape of biomedical engineering.

## Figures and Tables

**Table 1:** Examples of peptide sequences used to create biomaterials and their application in biomedicine.

Peptide Classification	Peptide Sequence	Structure/Formation	Biological Role/Application	Ref.
Ionic Peptides	EFK16-II	β-sheet	Glucose biosensing, biosensor design	[10]
	EAK16-II	β-sheet	Anticancer activity, biomaterial mimicking ECM	[11]
	RADA16	Nanofibers	Tissue engineering, hemostatic agent	[15,16]
Amphiphilic Peptides	C16IKPEAP/C16IKPEAPG	Micelles and fibrils	Gastrointestinal peptide hormone-related applications	[17]
	KLVFFAK	Amyloid 2D nanosheets	Antimicrobial hydrogels, biofilm prevention	[18]
	Ac-RKKWFW-NH2 (PAF26)	β-sheet hydrogel	Antimicrobial activity against Candida and S. aureus	[19]
	NAVSIQKKK	Lipopeptide hydrogel	Antibacterial, wound healing	[20]
Peptidomimetics	KLVFFAK (KKd-11) (D-amino acids)	Hydrogel	Antimicrobial efficacy, biofilm prevention	[21]
	Fc-FFRGD	Nanostructure and hydrogel	Biomimetic material for drug encapsulation	[22]
Cyclic Peptides	Lanreotide	Hydrogel	Treatment of acromegaly and tumours	[23,24]
	P6 and P9	Short cyclic peptides	Drug delivery to EGFR-targeted cells	[25]
Antimicrobial Peptides	Tet213	Collagen-integrated	Wound healing, antimicrobial	[26]
	Piscidin-1	Chitosan scaffold integration	Porous biomaterial, biodegradation	[27]
	LL-37	Conjugated onto collagen	Antibacterial against various pathogens	[28]
Dipeptides	Diphenylalanine (FF)	Nanotubes	Drug release, stable nanostructures	[29]

## Data Availability

Data sharing is not applicable.
